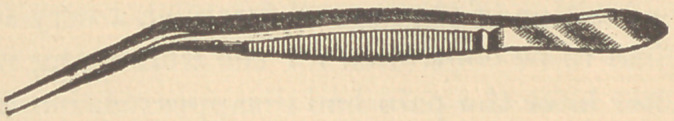# American Academy of Dental Science

**Published:** 1891-09

**Authors:** 

**Affiliations:** American Academy of Dental Science


### AMERICAN ACADEMY OF DENTAL SCIENCE.

THE regular monthly meeting of the American Academy of Dental Science was held in the Boston Medical Library Association rooms on May 6, 1891, President Seabury in the chair.

The paper for the evening was read by A. K. Stone, M.D. Subject, "Dental Responsibillty for Early Diagnosis of Tumors of the Mouth and Jaws."

(For Dr. Stone's paper, see p. 602.)

### DISCUSSION.

*Dr. Fillebrown.*—Mr. President, I desire to thank Dr. Stone for the very intelligent, instructive, and exhaustive paper which he has favored us with. I feel consoled by what the doctor says about the paucity of cases which present themselves. Such cases have always been of interest to me; in a sense I have been looking out for them. I thought that perhaps I had found many less than I ought to have found. It seems, however, that I have at least had my share. To add to the interest of this occasion, I have selected three cases and will present them for your consideration to-night.

CASE I.—Miss ——, aged thirty-five. Odontocele in left superior cuspid region. The patient was brought to me by her father, a physician, who had extracted her upper teeth preparatory to a set of artificial teeth. Tumefaction of the left side remained, and in regard to these conditions he desired my advice.

Upon examination with a steel probe I felt what was evidently enamel deeply embedded in the tissues. An operation revealed a fully-developed cuspid tooth lying almost horizontally in the jaw, the crown pointing forward. The removal of the tooth was followed by immediate recovery, and the mouth was soon in perfect condition for a plate.

Such a case must come to very many of us, and the only peculiarity about it was that it showed the value of special judgment as perfected by special practice. The gentleman who brought the patient (and she was his daughter) was a man of large medical knowledge and of considerable surgical ability, and yet he combated my position very strongly and would not admit even to the very last that there was a tooth in the mouth. He was sure that they had all been removed.

I remember another case which illustrates a tumor of the crackling egg-shell quality as described by Dr. Stone. I never quite understood the cause of it. The patient was a young man, and the only abnormal appearance about his mouth was the fact that the six upper teeth, involving the region from one cuspid to another, were a good deal crowded. There was considerable tumefaction there, and upon pressure over the tumor a crackling sound could be heard and a crackling sensation felt. The tumor would yield to pressure, and upon removal of the finger a depression remained for a time, but the original shape was regained. The patient's teeth were so crowded and irregular that he desired them out, and as this seemed best in his condition, I removed them. There was no discharge from the tumor and nothing unusual occurred. The tumor got well in a short time after the removal of the teeth. Whether the crowded teeth could have caused that condition, I do not know. A probe passed up through the socket of one of the extracted central incisors showed quite a large cavity which remained open for a good while, but finally was completely cured without further treatment. As soon as advisable the patient had artificial teeth, and as far as I know has had no further treatment.

Case II.—Mrs. ——, aged forty-five. Epulis arising from the pericementum of the left inferior second bicuspid mesial surface. Cured without operation.

This tumor had existed for some time, and when my advice was asked it filled the space between the bicuspids and protruded considerably into the mouth. Applications at various times had failed to destroy it or much restrict its growth. The patient was very nervous, and would not consent to anæsthesia or to any operation without it. I concluded to try constant pressure with styptics. I selected the persulphate of iron, and taking a pellet of cotton of suitable size to fill the space between the teeth, I coated it with the powder and packed it down upon the tumor. This was repeated at intervals of one or two days for about two weeks, the cotton being packed directly upon the tumor. The bulk of the growth disappeared upon the second application. Then, by following carefully to the starting-point of the tumor, I succeeded in effecting a complete cure in the two weeks, and without any operation involving the cutting of soft tissues or removal of bone.

CASE III.—Mr. ——, aged thirty-five. Tumor of upper jaw with suppuration. In February, 1889, this patient came to the Harvard Dental Hospital, accompanied by his physician. He showed himself to be intellectually bright and capable. His stature was diminutive, he being less than four and one-half feet tall. The upper frontal portion of his skull was well developed, but the superciliary ridge was very receding, not projecting at all beyond his eyelids, and the whole surface of the skull was very irregular.

The calcareous elements were quite largely in excess in his bones, in consequence of which his limbs had been broken some six times by slight causes. His muscular system was very well developed. His upper jaw was very small, much smaller in proportion than the under, the latter being quite prominent. This patient showed an extremely long, soft palate, the uvula and opening to the posterior nares being quite as low as the normal position of the glottis. On the whole, his mouth exhibited a unique appearance. His upper jaw presented some very large and long teeth, much swelling of the soft tissues, with copious suppuration. The teeth were loose, and the cuspids and bicuspids seemed to move together.

His case had been diagnosed as necrosis, but as there was entire absence of the characteristic discharge, and to my touch the teeth moved independently of the jaws, I came to the conclusion that the condition was abscess caused by the teeth, and that removal of them would effect a cure. The evident ankylosis of the teeth, the enlargement immediately behind and connected with the bicuspid, and the excessive size of the apex of the roots as shown by the probe left no doubt in my mind that we had to deal with an odontocele.

I was quite unable to convince the patient's medical adviser of the correctness of my diagnosis before the operation, hence I was obliged to await the result. There was no question as to the necessity or propriety of operating for him, as, whatever the condition, operation was the only way to obtain relief.

The patient was anæsthetized with nitrous oxide. Ether was immediately added and ether narcosis induced. An incision was made along the necks of the teeth extending as far as the boundaries of the tumors, and the teeth seized with forceps and the several bodies removed. These I show in this connection. The odontoma of the left side is an irregular mass, one and one-fourth inches long by three-fourths of an inch in thickness, consisting of a cuspid, first bicuspid, and an aborted second bicuspid and molar. The crown of the cuspid and first bicuspid are normal in form and development. The root of the cuspid is enormously developed, it being seven-eighths of an inch long, the portion near the apex one-half of an inch in diameter, and much curved backward. The root of the first bicuspid is also very large, and the surfaces of both are very irregular and rough from hypercementosis. The second bicuspid and molar are entirely rudimentary, a section showing the irregular formation of secondary dentine, with no pulp cavity remaining and hardly a trace of a formerly-existing one. The cuspid was separated from the rest of the tumor, and with no sign of pericementum. The other three teeth were fused into a single mass, and were nearly covered with what seems to be serumal calculus.

The tumor from the left side consists of three teeth,—cuspid, bicuspid, and molar,—and is very similar to the other. The cuspid and bicuspid crowns are normally developed, the cuspid root very long and large, and the root of the bicuspid also very large, and both very irregular. The molar is a little more regular in form than the one on the left, with quite a large pulp chamber with a branch corresponding to a root canal, the whole forming one connected mass and covered with calculus. Attached to the right bicuspid is a portion of the alveolar wall united with it by complete ankylosis. There is also a small portion of bone attached to the left cuspid. There is no sign of necrosis on either of the tumors. The patient made a good recovery.

In March, 1891, the same patient returned to the hospital with a chronic abscess discharging under his chin on a line with the bicuspid teeth, and nearly in the median plane, which had failed to yield to continued treatment. The location of the fistula had misled all who had observed it, consequently he had suffered from it for a considerable time, and he now came to see if his teeth had anything to do with it. He supposed that all his teeth had been removed. Upon probing I found roots present which were evidently causing the abscessed condition.

He was again anæsthetized with nitrous oxide. I attempted to remove the roots under the gas, but this was not readily accomplished, the brief narcosis not being sufficient. Ether was administered, and the roots removed. The left root proved to be a bicuspid with very excessive hypercementosis and with calcareous deposits; the right, an unusually large cuspid root. The specimens you see with the others.

In the two years since the first operation, his upper jaw has become absorbed until his mouth is about the size of that of a child five or six years old. It would measure less than one inch from one molar ridge to another. It was a surprise to every observer.

The patient has not reported since the last operation, but I feel there is no doubt of his complete recovery.

*Dr. Clapp.*—I have been very much interested in the paper of Dr. Stone, and also in the remarks of Dr. Fillebrown, and I am very glad that Dr. Stone has emphasized the fact that these tumors he speaks of occur so rarely. Now, I have entered on my twenty-first consecutive year of practice in Boston, during which time I have probably seen at least an average number of patients with other practitioners, and during this whole time I have never seen a single example of either of the classes of tumors that has been mentioned. I might say further, in reference to the liability of cancers occurring from ragged roots or ragged teeth in the mouth causing irritation, I did have a patient many years ago who had a tooth of this kind. There was at that time a slight irritation that had been caused by the rough edge of the tooth, and I advised its extraction. I never saw the case again, but I learned some time afterwards that the patient died from cancer of the tongue caused by that tooth.

*Dr. Codman.*—I cannot say much on this subject. I have been looking for such tumors all these years and have seen very few, only one or two in all my practice, and those not important enough to fix in my mind, and yet I do not believe I have overlooked them. I think that we are not liable to meet with tumors of the jaw outside of the hospitals, except such small ones as can hardly be called tumors. It is important that some person should take up the subject as a specialty because the average dentist sees so few cases, and certainly if I had a patient with anything of that sort I would refer him to a specialist rather than perform an operation myself. I have read volumes on the subject, seen hundreds of plates, and yet have never been acquainted with an important case. I don't remember seeing one that I could bring forward at this time to interest the society.

*Dr. Preston.*—I don't know that I can say much about tumors, unless that term includes what are usually called "gum-boils," and I

don't see that any one gives a cure for them. I have had three cases of that kind situated in the roof of the mouth. They were thick and sometimes as large as the ball of your thumb, and in my practice of over fifty-two years I have only met with three cases. I have never heard of a cure for them; they generally dry up and disappear themselves, and all that I can say is, remove the cause, which is generally a dead tooth, and the swelling will disappear.

*Dr. Smith.*—Of course the case which Dr. Preston speaks of must be alveolar abcess, induced, as he says, by a dead tooth, and I think the majority of practitioners are curing such cases now without removing the tooth. The track is always there, and the seat of the abscess can be reached without extracting the tooth.

I will relate a case in practice of a tumor that was not a tumor. It was a case of severe swelling on the left side of the face low down in the jaw. The patient went to a surgeon who is on the staff of one of the hospitals here in Boston for treatment. He examined her carefully in his office and pronounced it a tumor, and advised her to go to the hospital at once and have it removed. Not satisfied with that, and being naturally fearful of undergoing an operation, she consulted another surgeon. He was not quite so positive in his diagnosis of the case, yet he also thought it was a tumor, and said that she had better go to the hospital and prepare for an operation. She finally came to my office, and the condition of the face when I saw it was the same as when she visited these two surgeons. The swelling was very hard, and she could hardly open her mouth,—not enough to admit a mouth-mirror,—so the examination was conducted under difficulties. I found that a tooth had been extracted on the affected side, and on futher examination I decided that the fang of a wisdom tooth was still there. Pressure revealed no soreness either within or without the face. A fine probe was introduced and it passed down indefinitely, so to speak, and I came to the conclusion that the trouble was produced by this wisdom tooth. I advised its extraction, which was consented to, and with the aid of antiseptic washes and applications to the outside of the face that so-called-tumor disappeared. I mention this merely to show how, as Dr. Stone says in his paper, a physician could be mistaken in his diagnosis and call a swelling a tumor, when the trouble proceeded from a tooth. It is in such cases that the services of a dentist might frequently be of great service.

*Dr. Taft.*—I had recently a case in practice so analogous to that of Dr. Smith's that I will merely mention it. The patient presented herself one day with a very large swelling on the gum directly over the root of the left superior canine. It was then much smaller than it had previously been, but when she came to me her whole lip was so swollen that her face was much disfigured. The swelling was very hard, with no fluctuation. Not being sure just what the trouble was, I thought best to consult a physician. It seemed to me that it could not come directly from the canine root, it was so small, but of course I had it in mind to remove the root sooner or later. The physician who consulted with me pronounced the case a cyst, as I had thought it probably was. After an application of cocaine, the cyst was lanced, and a very thin and copious watery discharge came from it. The patient was given gas and the root removed, and the swelling subsequently disappeared. It seems to me that that case was not really a tumor, but a simple cyst. Having so few of these cases in practice, a correct diagnosis is often difficult unless we keep ourselves well informed in dental pathology. Cases of epulis are the ones which we are most likely to meet with in practice, and I recall, as a student, how difficult it was to distinguish them from a simple hypertrophied gum. I feel quite interested in the subject of epulis, although since leaving the Harvard Dental School not a single case has come to my practice. I remember seeing at the Infirmary many operations for their removal, and call to mind how vascular some of them were. I am quite interested to know whether they can be removed successfully by a simple cutting away to the alveolus and a thorough scraping of the process.

*Dr. Fillebrown.*—Mistaken judgment seems to be one of the subjects that has come up here, and it is always very easy for us to see other people's mistakes and overlook our own. I have no doubt that I make my share of them and that other people see them and criticise them. I have three cases in my mind that illustrate mistaken judgment. Some years ago a patient of mine was taken ill and had a swollen face, and went to a physician who treated him for erysipelas of the face, gave him a good long treatment, and brought him through to a splendid cure. Soon after this the patient came to me with a fistula on the gum in the region of the apex of the cuspid tooth. He had suffered from an alveolar abscess. The other two cases illustrate mistakes arising from over-confidence in specialists. One of them presented a large swelling in the cheek which a physician pronounced an alveolar abscess. A short examination proved to me that the swelling was not attached to the jaw, and I said to the patient, "This is simply an abscess in the soft tissues, and if you will continue poulticing it you will soon find relief." In speaking of this case later to a surgical friend, I explained why I considered it an abscess in the soft tissues and not an alveolar abscess,—namely, because it was entirely free from the jaw. Some years afterwards my surgical friend was called upon to treat a man who had a swelling in the right inferior jaw. He passed his finger in, and decided that the swelling was not connected with the jaw, and told the patient that it was a tumor and ought to be taken out at once. A few days later the patient came to me, and I saw that without a doubt his trouble was an alveolar abscess due to a first inferior molar, and it was as markedly connected by a fistulous canal with that molar as in any case I ever saw. The surgeon is surely as bright as any in New England and yet he was in error. After a little treatment in connection with the tooth the tumor got well.

A good illustration of epulis presented itself at the dental hospital the other day. The patient came for the extraction of teeth, and after they had been removed there was to be seen floating about in the mouth an epulis fully as large as the end of my thumb, and attached to the jaw by a little slender cord not one-sixteenth of an inch in diameter, but quite long.

*Dr. Eames.*—It is quite easy to pronounce a swelling in the jaw a tumor, but not so easy to classify it. There is a good deal of confusion with regard to epulis. I should like to know whether the cases spoken of here to-night were submitted to a microscopical examination; if not, I should reserve my judgment with regard to two of them. The term epulis indicates nothing as to the nature of the growth, and it would seem better to drop the name altogether and adopt some term which would indicate in a degree the origin and structure of the growth. The treatment should vary in accordance with our conception of the disease. If it is a tumor originating in the periosteum or endosteum of the alveolar process, then the alveolar process and a considerable portion of the jaw should be removed. I agree with Dr. Taft as to the difficulty of diagnosis. Quite a large growth may come from the irritation of a deposit of tartar. Three years ago I treated such a case by simple excision, and there has been no return up to the present time. On finding a swelling, we as dentists should first look for a foreign body or irritating cause, and attempt its removal. If there is not a rapid cure following local treatment, we should submit the growth to the microscope as suggested by Dr. Stone in his paper. Thus the case can be diagnosed more clearly and treated more intelligently.

*Dr. Stone.*—I don't know that I have anything to add to what I have already said, but the point that tumors of the jaw are rare I think will be borne out by surgeons as well as by members of your society. I have under observation rather an interesting case of an apparently healthy young woman between twenty-five and thirty years of age, who some time ago was attacked with a severe pain about the position of the mental foramen, a very severe neuralgic pain that had to be controlled for the time being with morphia. When I saw her later the pain had disappeared, but there was no feeling in the teeth on one side as far as the centre of the jaw, and there was no feeling in the lip and skin outside. Upon the jawbone, not in the alveolus, was a swelling about the size of a walnut; it was evidently a new growth inside the jawbone which had pressed upon the nerve and caused the pain, and finally the destruction of the nerve and consequent paralysis. The nomenclature of tumors has been changing so rapidly in the last years that it is difficult to agree upon a precise meaning for a given term. The word "tumor" is a general name, meaning swelling, and "epulis" means a new growth from the gums. The diagnosis and consequent operative procedure will have to be governed by what the pathologist may decide in a given case.

Subject passed.

*Dr. George T. Baker.*—I have here a simple device which I use to insert a tooth temporarily in the mouth in case one has been extracted. It is only the work of a few minutes. It consists of a yoke of thin platinum wire, the two arms of which bend over the teeth adjoining the space to be filled. A plate tooth is ground to fit the gum and rest upon the yoke. The tooth is backed with platinum and soldered, and finally tied in place with floss silk. When properly tied it is very firm and serviceable.

*Dr. Fillebrown.*—Here is a piece of jawbone brought to me by a patient, and taken from the Florida mounds. Some of the teeth have been broken off, but two of them are whole. The broken ones show a perfect quality. The owner of this jaw must have lived several hundred years ago, for it is partially petrified.

*Dr. Meriam.*—I have always had trouble in finding a suitable clamp for lower bicuspids where the molars were lost, owing to the rubber tipping the clamp forward. Last fall I asked Dr. Ivory to make me one; he has followed directions carefully, and the clamp I show is the best I have used. The bow goes up at nearly right angles, and the leverage is but slight. Mr. Knapp offers to keep them if there is a demand. I show also a pair of tweezers. I

cannot call them original, as since I had them made I have found a similar but stouter form in some of the trade catalogues. Mine must therefore be called a modification, and will be found useful in reaching into deep cavities. They are short enough to be easily used at any angle. The cut I shall introduce shows them at half-size.

I had them made by Mr. Goldthwaite, on Washington Street, where they can be had, or ordered through any druggist or dealer in surgical instruments.

William H. Potter, D.M.D.,
*Editor American Academy of Dental Science.*

## Figures and Tables

**Figure f1:**